# Draft Genome Sequence of Bacillus salarius IM0101, Isolated from Hypersaline Soil in Inner Mongolia, China

**DOI:** 10.1128/MRA.01686-18

**Published:** 2019-01-31

**Authors:** Wariya Yamprayoonswat, Supichaya Boonvisut, Watthanachai Jumpathong, Satapanawat Sittihan, Pattarawan Ruangsuj, Suwanan Wanthongcharoen, Nuttida Thongpramul, Salisa Pimmason, Bo Yu, Montri Yasawong

**Affiliations:** a Environmental Toxicology, Chulabhorn Graduate Institute, Bangkok, Thailand; b Center of Excellence on Environmental Health and Toxicology (EHT), Office of Higher Education, Bangkok, Thailand; c Chemical Biology, Chulabhorn Graduate Institute, Bangkok, Thailand; d CAS Key Laboratory of Microbial, Physiological, and Metabolic Engineering, Institute of Microbiology, Chinese Academy of Sciences, Beijing, China; Georgia Institute of Technology

## Abstract

Bacillus salarius IM0101 is a halophilic bacterium that was isolated from soil in Inner Mongolia, China. The genome sequence of B. salarius IM0101 contains a biomarker gene for polyhydroxyalkanoate (PHA) synthesis. This 6.9-Mb draft genome sequence of *B. salarius* IM0101 will provide new insights into the organism’s PHA production machinery.

## Abstract (precis)

Bacillus salarius IM0101 is a halophilic bacterium that was isolated from soil in Inner Mongolia, China. The genome sequence of B. salarius IM0101 contains a biomarker gene for polyhydroxyalkanoate (PHA) synthesis.

## ANNOUNCEMENT

Bacillus salarius is Gram-positive, rod-shaped, and strictly aerobic bacterium. The optimum growth conditions are 10 to 12% (wt/vol) NaCl, 30°C, and pH 8.0. MK-7 is its major menaquinone. B. salarius contains saturated branched fatty acids, including anteiso-C15:0 and anteiso-C17:0. The cell wall of B. salarius is A1γ type peptidoglycan with meso-diaminopimelic acid, similar to most of the members of the genus *Bacillus*; also similar to other *Bacillus* species are the components of major fatty acids, lipoquinone, and G+C content (1).

Strain IM0101 was isolated from soil collected in Bayan Nur, Inner Mongolia Autonomous Region, China. The soil sample contained 18% (wt/vol) NaCl. A single colony of B. salarius IM0101 was cultured in JCM169 broth (2) for 3 days at 37°C with shaking at 250 rpm. The partial 16S rRNA gene (1,353 bp) of strain IM0101 was identified using the EzBioCloud database (3) and was 99.93% identical to that of Bacillus salarius. High-quality genomic DNA (gDNA) of B. salarius IM0101 was obtained using the phenol-chloroform method described by Sambrook and Russel (4). The quantity of gDNA was determined by NanoDrop spectrophotometry (Thermo Fisher Scientific, USA). A sequencing library was prepared using the Ion Plus fragment library kit and the Ion PI Hi-Q OT2 200 template kit. The sequencing was performed on the Ion Proton sequencer using the Ion PI Hi-Q sequencing 200 kit and the Ion PI chip (Thermo Fisher Scientific, USA). The average length of the reads was 145 bp. There were 10,229,665 raw reads (208× depth of coverage) generated from the sequencing run.

*De novo* assembly of the raw reads was performed using SPAdes version 3.12.0 (5). The quality of the genome assemblies was determined using QUAST version 5.0.0 (6). The draft genome sequence of B. salarius IM0101 consists of 6,960,367 bp in 326 contigs with an *N*_50_ value of 112,419 bp and a CG content of 40.1%. Genome annotation and gene prediction were performed using Prokka version 1.13.3 (7). The predicted genome sequence of B. salarius IM0101 contains 6,970 protein-coding sequences and 98 tRNA, 5 rRNA, and 2 transfer-messenger RNA (tmRNA) genes.

There is one copy number of the *phaC* gene in the B. salarius IM0101 genome. The *phaC* gene encodes the PhaC subunit of polyhydroxyalkanoate (PHA) synthase, the key enzyme of PHA production (8–10).

### Data availability.

The whole-genome shotgun sequence of Bacillus salarius IM0101 has been deposited at DDBJ/ENA/GenBank under the accession number RBVX00000000 (version RBVX01000000) and SRA accession number SRR8357421.
